# Autonomic markers of emotional processing: skin sympathetic nerve activity in humans during exposure to emotionally charged images

**DOI:** 10.3389/fphys.2012.00394

**Published:** 2012-10-01

**Authors:** Rachael Brown, Cheree James, Luke A. Henderson, Vaughan G. Macefield

**Affiliations:** ^1^School of Medicine, University of Western SydneySydney, NSW, Australia; ^2^Neuroscience Research AustraliaSydney, NSW, Australia; ^3^Department of Anatomy and Histology, University of SydneySydney, NSW, Australia

**Keywords:** skin sympathetic nerve activity, emotionally charged images, microneurography, sweat release, skin blood flow

## Abstract

The sympathetic innervation of the skin primarily subserves thermoregulation, but the system has also been commandeered as a means of expressing emotion. While it is known that the level of skin sympathetic nerve activity (SSNA) is affected by anxiety, the majority of emotional studies have utilized the galvanic skin response as a means of inferring increases in SSNA. The purpose of the present study was to characterize the changes in SSNA when showing subjects neutral or emotionally charged images from the International Affective Picture System (IAPS). SSNA was recorded via tungsten microelectrodes inserted into cutaneous fascicles of the common peroneal nerve in ten subjects. Neutral images, positively charged images (erotica) or negatively charged images (mutilation) were presented in blocks of fifteen images of a specific type, each block lasting 2 min. Images of erotica or mutilation were presented in a quasi-random fashion, each block following a block of neutral images. Both images of erotica or images of mutilation caused significant increases in SSNA, but the increases in SSNA were greater for mutilation. The increases in SSNA were often coupled with sweat release and cutaneous vasoconstriction; however, these markers were not always consistent with the SSNA increases. We conclude that SSNA, comprising cutaneous vasoconstrictor and sudomotor activity, increases with both positively charged and negatively charged emotional images. Measurement of SSNA provides a more comprehensive assessment of sympathetic outflow to the skin than does the use of sweat release alone as a marker of emotional processing.

## Introduction

The complexities of human emotion, in particular negative emotion or mental stress have been widely studied, with physiological responses such as blood pressure, heart rate, cutaneous blood flow, and sweat release commonly being measured during cognitive stress (such as the Stroop color-word conflict test or mental arithmetic) and perceived stress (viewing of negatively charged emotional images) (Hare et al., 1970; Frijda, 1986; Ellsworth, 1991; Callister et al., 1992; Fox, 2002; Kern et al., 2002; Carter et al., 2008). In addition, some studies have used intraneural microelectrodes (microneurography) to record muscle sympathetic nerve activity (MSNA) and skin sympathetic nerve activity (SSNA) during emotions (Delius et al., 1972a,b; Hallin and Torebjork, 1974; Callister et al., 1992; Carter et al., 2005, 2008), although the results of these investigations are varied. While the use of mental arithmetic, the Stroop color-word test and the viewing of negatively charged images are extensively used to evoke changes in sympathetic activity, the viewing of positively charged images has received less attention. Only one study has examined the effects of MSNA while viewing negatively charged images (Carter et al., 2008), with no reported studies on the effect of SSNA while viewing any emotionally charged images (negative or positive).

While most investigations exploring sympathetic innervation of the skin focus on its role in thermoregulation, the system has also been implicated in emotional processing and is heightened during peak emotional states. However, the majority of studies examining emotionally charged stimuli have utilized sweat release to infer increases in sympathetic outflow, which has been shown to have a poor relationship with SSNA (Kunimoto et al., 1992). Therefore, the aim of the present study was to characterize the changes in SSNA using microneurography while showing subjects neutral or emotionally charged images from the International Affective Picture System (IAPS), which is a widely recognized stimulus system that is used to study the effects of emotion on human subjects (Lang et al., 1997). Our aim was to compare effector organ responses such as blood pressure, heart rate, respiration, and in particular sweat release and cutaneous blood flow, with direct microneurographic recordings of SSNA. The intention was to elicit the negative emotion of disgust via images of mutilation and injury, while the positive emotion of pleasure was elicited via erotic images.

## Methods

### General procedures

Studies were performed on five male and five female healthy subjects (age 20–46 years). The studies were conducted under the approval of the Human Research Ethics Committee of the University of Western Sydney, and satisfied the Declaration of Helsinki. Each subject gave informed written consent before participating in the study, and was told that they could withdraw from the experiment at any time. Subjects reclined comfortably in a chair in a semi-recumbent position with legs supported horizontally. Care was taken to ensure a calm and quiet environment to minimize spontaneous arousal responses. A comfortable ambient temperature was also maintained (22°C), as sympathetic outflow to the skin is susceptible to changes in ambient temperature. ECG (0.3–1.0 kHz) was recorded with Ag–AgCl surface electrodes on the chest, sampled at 2 kHz, and stored on computer with other physiological variables using a computer-based data acquisition and analysis system (PowerLab 16SP hardware and LabChart 7 software; ADInstruments, Sydney, Australia). Blood pressure was recorded continuously using finger-pulse plethysmography (Finometer Pro, Finapres Medical Systems, The Netherlands) and sampled at 400 Hz. Respiration (DC-100 Hz) was recorded using a strain-gauge transducer (Pneumotrace, UFI, Morro Bay CA, USA) wrapped around the chest. Changes in skin blood volume, reflecting changes in skin blood flow, were monitored via a piezoelectric transducer applied to the pad of a finger; from this signal pulse amplitude was calculated using the Cyclic Measurements feature in the LabChart 7 software. A decrease in pulse amplitude was used to indicate a decrease in skin blood flow. Skin potential (0.1–10 Hz; BioAmp, ADInstruments, Sydney, Australia) was measured across the palm and dorsum of the hand; changes in skin potential reflect sweat release.

### Microneurography

The common peroneal nerve was located at the fibular head by palpation and superficial electrical stimulation through a surface probe (3–10 mA, 0.2 ms, 1 Hz) via an isolated constant-current source (Stimulus Isolator, ADInstruments, Sydney, Australia). An insulated tungsten microelectrode (FHC, Maine, USA) was inserted percutaneously into the nerve and manually advanced toward a cutaneous fascicle of the nerve while delivering weak electrical pulses (0.01–1 mA, 0.2 ms, 1 Hz). An uninsulated subdermal microelectrode served as the reference electrode and a surface Ag–AgCl electrode on the leg as the ground electrode. A cutaneous fascicle was defined as such if intraneural stimulation evoked paraesthesiae without muscle twitches at stimulation currents at or below 0.02 mA. Once a cutaneous fascicle had been entered, neural activity was amplified (gain 10^4^, bandpass 0.3–5.0 kHz) using a low-noise, electrically isolated, headstage (NeuroAmpEx, ADInstruments, Sydney, Australia). The identity of the fascicle was confirmed by activating low-threshold mechanoreceptors—stroking the skin in the fascicular innervation territory. The position of the microelectrode tip was then adjusted manually until spontaneous bursts of SSNA were identified. For identification purposes, individual bursts of SSNA were generated by asking the subject to take a brisk sniff or, with the subject's eyes closed, delivering an unexpected stimulus—such as a tap on the nose or a loud shout. Neural activity was acquired (10 kHz sampling) and sympathetic nerve activity was displayed as an RMS-processed (root mean square, moving average time-constant 200 ms) signal and analyzed on computer using LabChart 7 software.

### Emotional stimuli

Emotional state changes were produced by viewing standard images from the IAPS (Lang et al., 1997). Each picture used in the system has been extensively tested and rated for valency (its subjective impact ranging from extremely negative to extremely positive) and arousal. In our study, positive emotions were evoked by viewing images of erotica with high positive valence ratings, while negative emotions were evoked by viewing images of mutilation with high negative valence; both sets had high arousal ratings. Once a suitable intraneural site with spontaneous SSNA was found and the subject was relaxed, a 2-minute resting period was recorded, following which the subject was shown 30 neutral images, each image lasting 8 s, for a total of 4 min. This was followed by a block of 15 images (either erotica or mutilation) lasting 2 min. Images of erotica or mutilation were presented in a quasi-random fashion at a time unknown to the subjects, with each 2-minute block of emotionally charged images following a 2-minute block of neutral images. In total, each subject viewed 3 blocks of erotica and 3 blocks of mutilation with 6 intervening blocks of neutral images.

### Analysis

Peak amplitudes of SSNA, measured over consecutive 1-s epochs, coupled with the total number of sympathetic bursts, were measured over each 2-minute block. Visual inspection, coupled with auditory recognition of the neural signal, was used to identify individual bursts of SSNA. In addition, baseline was defined manually in the RMS-processed signal and the computer calculated the maximum amplitude above baseline. A beat-beat analysis was conducted for heart rate, blood pressure, skin blood flow, skin potential, and respiratory rate over each 2-minute block and a mean value for each block in each subject was derived. A mean group value for each 2-minute block could then be calculated and absolute changes derived. Absolute changes in skin potential and skin blood flow were normalized to the individuals average resting value. In addition to absolute changes for each 2-minute block, relative changes normalized to neutral were calculated for the resting period and positive and negative images. Repeated Measures Analysis of Variance of each physiological parameter across the three stimulus conditions, coupled with a Newman–Keuls test for multiple comparisons, was used for statistical analysis of the data (Prism 5 for Mac, GraphPad Software Inc, USA). In addition, paired *t*-tests were used to compare relative changes (normalized to neutral) in various physiological parameters for the erotica and mutilation data sets. The level of statistical significance was set at *p* < 0.05.

## Results

**Figure 1:**
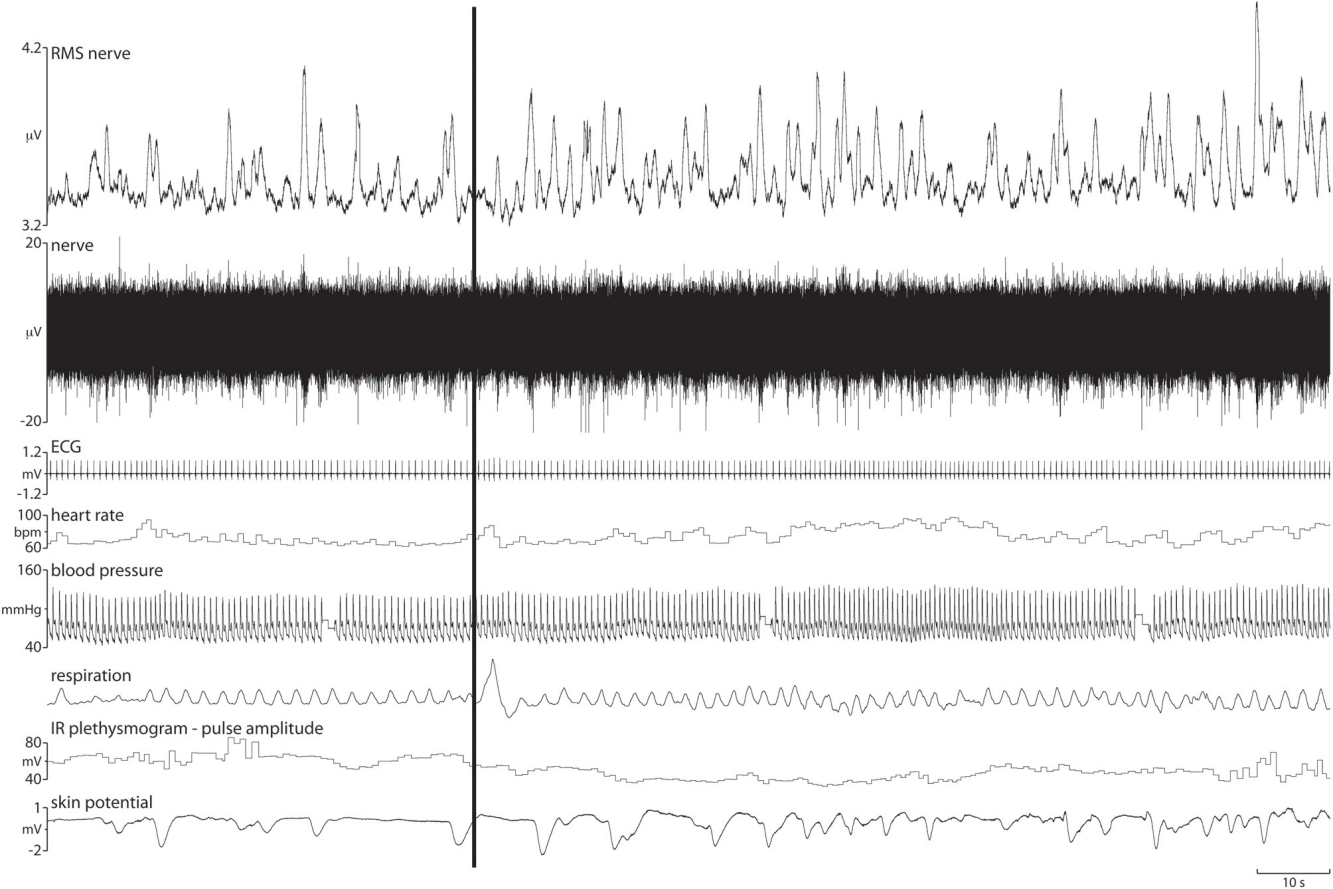
**Raw and calculated data obtained from a female subject during exposure to a 2-minute block of mutilation.** Both intraneural and physiological parameters are shown. The vertical black bar represents the start of the mutilation blosck, with a 1-minute period of neutral images preceding this. An increase in both burst frequency and amplitude of skin sympathetic nerve activity can be seen in the 2-minute block of negatively charged images relative to the preceding neutral. Cutaneous vasoconstriction and sweat release can also be seen, but the responses are sluggish and do not correlate well with the burst of sympathetic activity.

Figure 1 shows raw and calculated data obtained from a 21 year-old female during the last minute of presentation of a 2-minute block of neutral images, and the entire 2-minute block of negatively charged images. It can be seen that there were no overt changes in blood pressure or heart rate when viewing images of mutilation, relative to viewing neutral images, yet SSNA clearly did increase, both in burst amplitude and frequency. Moreover, as expected, cutaneous vasoconstriction and sweat release occurred. However, it is apparent that the latter indirect measures of skin sympathetic outflow are sluggish and long-lasting, correlating poorly with the actual bursts of SSNA that bring about the cutaneous vasoconstriction and sweat release. Respiration, known to influence SSNA, also showed no overall change in either depth (amplitude) or rate.

On the whole, neither blood pressure, heart rate, cutaneous blood flow or sweat release showed any significant changes during viewing of emotionally charged images, compared to viewing neutral images. Changes in respiration were variable, with increases in respiration rate or amplitude occasionally being seen, but this was predominantly in females when exposed to negatively charged images; again, however, these changes were not statistically significant.

**Figure 2:**
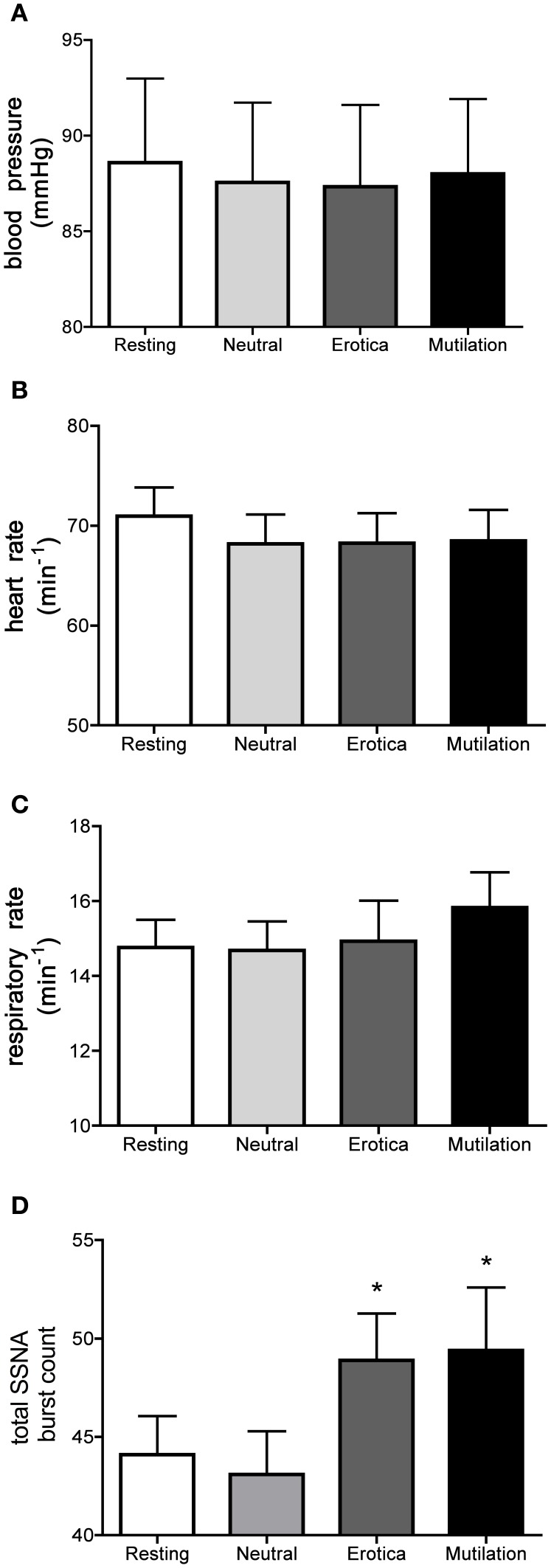
**Mean ± SE absolute values of blood pressure (A), heart rate (B), respiratory rate (C) and total burst count of skin sympathetic nerve activity (D) across the four conditions.** As can be seen, no statistical differences exist except for the SSNA burst count. Erotica and mutilation were statistically different from both resting and neutral. ^*^p < 0.05. Abbreviations: mmHg = millimetres of mercury.

Absolute values for blood pressure, heart rate, respiratory rate and total SSNA burst count across rest (no images), when viewing neutral images and when viewing images of erotica or mutilation, are illustrated in Figure 2. Only SSNA showed significant changes across the four conditions: there were no differences in total burst count at rest and when viewing neutral images, but significant increases when viewing either images of erotica or mutilation. However, there were no significant differences in the magnitude of these increases in the two conditions.

**Figure 3:**
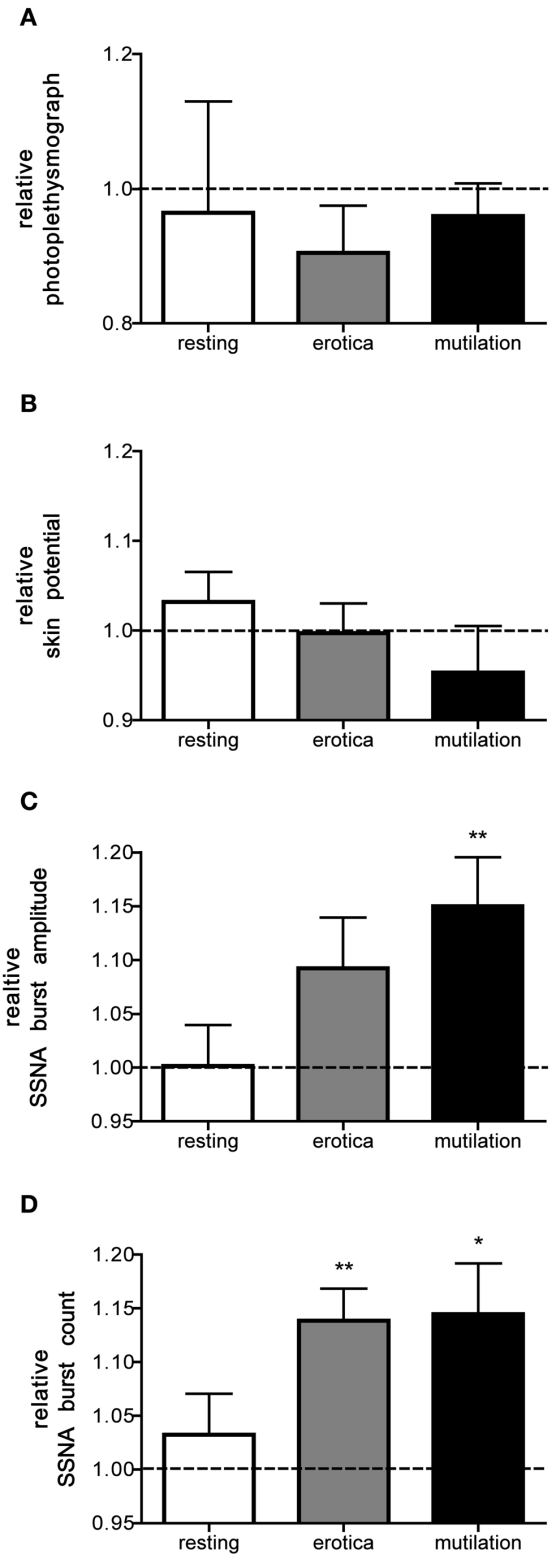
**Mean ± SE relative changes in skin blood flow (A), sweat release (B), burst amplitude (C) and frequency of skin sympathetic nerve activity (D), for the resting period, positive images, and negative images, all normalized to the neutral condition.** Statistical differences are only seen for SSNA burst amplitude (mutilation) and SSNA burst count (erotica and mutilation). The dashed line represents the neutral value. Statistics refer to differences from neutral. ^*^p < 0.05, ^**^p < 0.01.

Figure 3 shows relative changes in skin blood flow, sweat release, and SSNA total burst amplitude and frequency—normalized to levels recorded when viewing neutral images—in the resting state and when viewing the emotionally charged images. While significant increases in the number of SSNA bursts were seen for both positively charged and negatively charged images, increases in burst amplitude only reached statistical significance for the images of mutilation.

## Discussion

This study has shown for the first time that viewing blocks of emotionally charged images results in a significant increase in SSNA, measured as total burst count as well as burst amplitude, yet no significant changes in other physiological parameters, such as blood pressure, heart rate, or respiration. Moreover, that there were no significant changes in skin blood flow or sweat release indicates that recording the nerve activity directly provides a more sensitive means of assessing sympathetic outflow to the skin than does measurement of indirect markers of cutaneous sympathetic activity. Overall, similar increases in SSNA were evoked for both positively and negatively charged emotional stimuli, suggesting that increases in SSNA can be evoked by visual emotional stimuli (regardless of valence).

Indirect measurements of the sympathetic nervous system in response to emotion have been widely utilized, with picture viewing, film clips, personalized recall, and threat of shock being just some of the methods used to elicit a wide range of emotions (Lang et al., 1993; Christie and Friedman, 2004; Blechert et al., 2006; Rochman and Diamond, 2008). While cardiovascular, respiratory, and electrodermal measures can be useful indicators of the state of the sympathetic nervous system, the physiological responses reported in previous studies were variable, and the exclusive use of such measures can be misleading. In addition to the varied results, the comparison of studies can be difficult as different studies often utilized a variety of methodologies and physiological measurements. The IAPS is one method used in an attempt to elicit emotions such as disgust and anticipatory pleasure. Yet this standardized approach can still yield conflicting results: Lang et al. (1993) found an increased skin conductance response (i.e., an increase in sweat release) when viewing erotic images, while Ritz et al. (2005) reported little to no change in this same parameter. Furthermore, while viewing mutilation-related images of disgust, Lang et al. (1993) observed a decrease in heart rate and an increase in skin conductance yet, despite using images of similar valence and arousal scores, Ritz et al. (2005) found an increase in heart rate and no change in skin conductance. Increased respiratory cycle time while viewing images of disgust has also been reported (Ritz et al., 2005), yet Sokhadze (2007) observed no changes in respiration in the same context. Likewise, Christie and Friedman (2004) and Kunzmann et al. (2005) reported increased blood pressure in response to film clips of disgust, while Rohrmann and Hopp (2008) observed no changes in blood pressure while presenting similar film clips.

This variation in effector-organ responses to emotional stimuli that share similar valence and arousal scores emphasizes the need for direct measurements of sympathetic nerve activity. While microneurographic studies have been conducted that have directly recorded sympathetic nerve traffic in response to emotional stimuli, the results are varied and usually involved evoking a cognitive stress (mental arithmetic) rather then an emotional stress (Hallin and Torebjork, 1974; Callister et al., 1992; Carter et al., 2005). However, Carter et al. (2008) did examine the effects of presenting negatively charges images from the IAPS set on sympathetic outflow to muscle, but found no increases in either sympathetic nerve activity or any other physiological responses. This is, in part, in agreement with the present study: we also found no physiological responses to negatively charged images. In spite of this, there are no reported studies on the effects of viewing any emotionally charged images (negative or positive) on sympathetic outflow to *skin*.

We know that bursts of SSNA directed to hairy skin are composed of cutaneous vasoconstrictor and sudomotor impulses, and that arousal leads to co-activation of these cutaneous vasoconstrictor and sudomotor neurones, as evidenced by the early studies of Delius and colleagues (1972b) and Hagbarth and colleagues (1972). These studies also found that arousal stimuli can induce bursts of SSNA that are highly responsive, but with no correlation to blood pressure changes or any coupling to heart rate. These studies, however, used cognitive stress and threat of shock to induce an increase in sympathetic outflow, unlike the present study that used passive viewing of emotional images. Nonetheless, like these studies, the present study found that not only were bursts of SSNA highly responsive to emotional stimuli, but also these responses could be accompanied by complex vasomotor and sudomotor responses that often had a long delay that did not correlate with the bursts of SSNA and were sluggish in nature.

## Limitations

We used 2-minute blocks of stimuli, in order to induce a state of emotional engagement, but it is possible that such a long period may have diluted some of the physiological responses. However, this would be expected to have a similar effect on the direct nerve recordings, yet this appeared not to be the case. While emotionally charged images are arousal stimuli that would expect to exert emotionally generated physiological responses, one needs to take into account the varied background of subjects—including temperament and personality. The majority of subjects included in the current study consisted of young individuals, with some young females being naïve to the images of erotica, while others were not. This will impact on the degree of responses across the subjects. In addition, viewing of negatively charged images may have very little impact on those who have previously been exposed to such images or circumstances, and examining the effects of such images on those with exposure and those who are naïve would be an interesting comparative study.

Another limitation of studying the physiological effects of emotionally charged images is the use of neutral images in between blocks of emotionally charged images. While the valency of these neutral images is low, there will be a greater response in some individuals depending upon the image viewed. For example, viewing an image of an airplane or airport in individuals who have a fear of flying may evoke responses similar to those produced by viewing negatively charged images. Nevertheless, on average, there were no differences in levels of SSNA when viewing the set of neutral images compared to when subjects were just relaxed and not viewing any images (i.e., the rest period that preceded delivery of the sequences of visual stimuli).

## Conclusions

We conclude that SSNA, comprising cutaneous vasoconstrictor and sudomotor activity, increases with both positively charged and negatively charged emotional images. Using intraneural microelectrodes to record directly from postganglionic sympathetic axons directed to the skin, we have revealed responses to viewing emotionally charged images that provide a more comprehensive assessment of sympathetic outflow to skin than does recording indirect markers of skin sympathetic outflow—sweat release or skin blood flow.

### Conflict of interest statement

The authors declare that the research was conducted in the absence of any commercial or financial relationships that could be construed as a potential conflict of interest.
